# Physical activity level and influencing factors in pediatric Wolff-Parkinson-White syndrome patients: A case–control study

**DOI:** 10.1007/s00431-025-06290-7

**Published:** 2025-07-09

**Authors:** Haluk Tekerlek, Emine Burcu Özcan, Tuğba Çetin, Melda Sağlam, Naciye Vardar-Yağlı, Tevfik Karagöz, İlker Ertuğrul

**Affiliations:** 1 Department of Cardiopulmonary Physiotherapy and Rehabilitation, Faculty of Health Sciences, Karamanoglu Mehmetbey University, Karaman, Türkiye; 2 Department of Cardiorespiratory Physiotherapy and Rehabilitation, Faculty of Physical Therapy and Rehabilitation, Hacettepe University, Ankara, Türkiye; 3 Department of Pediatrics, Faculty of Medicine, Hacettepe University, Ankara, Türkiye; 4 Department of Pediatric Cardiology, Faculty of Medicine, Hacettepe University, Ankara, Türkiye

**Keywords:** Arterial stiffness, Cardiopulmonary exercise test, Physical activity, Pediatrics, Wolff Parkinson-White

## Abstract

This study aimed to evaluate physical activity levels and identify factors influencing physical activity in paediatric Wolff-Parkinson-White (WPW) syndrome patients. The clinical characteristics, arterial stiffness assessed using a non-invasive oscillometric method, and cardiorespiratory fitness measured through cardiopulmonary exercise test were recorded. Physical activity levels using Physical Activity Questionnaire for Older Children (PAQ-C), outcome expectations, perceptions of family support, as well as the barriers reported by participants’ parents, were evaluated using standardized questionnaires. Twenty-six paediatric WPW patients (11.12 years, 7 female) and 19 controls (12.31 years, 6 female) were included, with similar age, gender, and body composition (*p *> 0.05). Peak oxygen uptake (VO₂_peak_), peak heart rate (HR_peak_), and HR reserve (HRR) were significantly lower in the WPW group compared to controls (*p* < 0.05). Pulse wave velocity (PWV) was similar between both groups. Although patients tended to have lower PAQ-C scores compared to their controls (*p* = 0.097), scores for negative and positive physical activity outcome expectancies, perceived home support for physical activity (Physical Activity Home Environment), and parents’ perceived barriers to physical activity including both environmental and personal domains (Physical Activity Barriers) were similar between groups (*p* > 0.05). In WPW patients, PAQ-C scores correlated positively with VO₂_peak_ (*r* = 0.495; *p* = 0.010) and negatively with PWV (*r* = −0.453; *p* = 0.020). While paediatric WPW patients and their healthy peers show similar physical activity levels, expectations, family support, and parental barriers, their lower cardiorespiratory fitness may increase the risk of reduced physical activity. These findings suggest a link between habitual physical activity, exercise capacity, and vascular health, emphasizing the complex interplay of physiological and psychosocial determinants of physical activity in WPW.

*Trial registration*: NCT06349109 (2024-03-30

**Table:** 

**What is Known:** • *Children with heart disease generally exhibit lower physical activity levels compared to their healthy peers.* • *Both physiological and psychosocial factors are known to influence physical activity levels in children with heart disease.*	
**What is New:** • *Physical activity levels, outcome expectancies, family support, and perceived parental barriers were similar between pediatric WPW patients and healthy controls.* • *Lower exercise capacity and altered vascular health parameters may contribute to a higher risk of reduced physical activity in children with WPW syndrome.* • *These findings highlight the importance of addressing both physiological and psychosocial factors when promoting physical activity in pediatric WPW patients.*

**What is Known:**

• *Children with heart disease generally exhibit lower physical activity levels compared to their healthy peers.*

• *Both physiological and psychosocial factors are known to influence physical activity levels in children with heart disease.*

**What is New:**

• *Physical activity levels, outcome expectancies, family support, and perceived parental barriers were similar between pediatric WPW patients and healthy controls.*

• *Lower exercise capacity and altered vascular health parameters may contribute to a higher risk of reduced physical activity in children with WPW syndrome.*

• *These findings highlight the importance of addressing both physiological and psychosocial factors when promoting physical activity in pediatric WPW patients.*

## Introductıon

Wolff-Parkinson-White (WPW) syndrome is characterized by an unusually short PR interval, the presence of a delta wave on the electrocardiogram (ECG), and/or episodes of tachyarrhythmias such as supraventricular tachycardia or atrial fibrillation [1, 2]. This syndrome arises from an accessory conduction pathway that allows premature excitation of the ventricles, potentially leading to serious arrhythmic events, including atrial fibrillation, ventricular fibrillation, cardiac arrest, and sudden cardiac death in certain cases [2].

Physical activity is crucial for cardiovascular health, yet it is well-established that children with heart disease generally exhibit lower physical activity levels compared to their healthy peers [3–5]. The reasons behind low physical activity levels in patients with WPW are not well-documented in the literature, but several factors may play a role. Cardiac conditions, in particular, can induce both structural and functional alterations in autonomic regulation, potentially accelerating disease progression and increased the risk of arrhythmias [6]. Moreover, cardiovascular autonomic dysfunction can adversely affect vascular function, especially in children with heart disease [7]. Additionally, decreased aerobic exercise capacity may further limit participation in physical activity [8]. In this context, cardiopulmonary exercise testing (CPET) and measures of arterial stiffness offer valuable insights into cardiorespiratory fitness and vascular health, serving as noninvasive tools for risk stratification in patients with WPW [9, 10].

Beyond these physiological limitations, psychosocial factors such as exercise restrictions, parental overprotection, and fear of physical activity have been shown to influence activity levels in children with heart disease [4, 11, 12]. A recent study on barriers and facilitators of physical activity in pediatric congenital heart disease populations highlighted the roles of both non-modifiable factors, such as age and gender, and modifiable factors, including anxiety, self-efficacy, and environmental influences [13].

Despite growing interest in promoting physical activity among children with heart disease, few studies have specifically addressed these issues in pediatric WPW patients. Understanding the determinants of physical activity in this group is essential for developing targeted interventions. Therefore, the aim of this study is to evaluate physical activity levels and to identify physiological and psychosocial factors associated with physical activity participation in pediatric patients with WPW syndrome.

## Methods

### Study design and participants

This case–control study was conducted at Hacettepe University between March and December 2024, in collaboration with the Pediatric Cardiology Department of the Medicine Faculty, and the Cardiorespiratory Physiotherapy and Rehabilitation Department of Physical Therapy and Rehabilitation Faculty. The study was approved by Hacettepe University Ethical Board (SBA 23/015) and registered on ClinicalTrials.gov (NCT06349109). Informed consent was obtained from all parents, and assent was provided by the children. The study protocol complies with the ethical guidelines of the 1975 Declaration of Helsinki.

A total of 26 pediatric patients diagnosed with WPW syndrome [mean age = 11.12 years (95% CI: 9.94–12.29); 7 females, 26.9%] and 19 age- and sex-matched healthy controls [mean age = 12.31 years (95% CI: 11.05–13.56); 6 females, 31.6%] were included in the study. Participants in the WPW group were aged between 7 and 18 years and were under follow-up at the pediatric cardiology clinic.

Inclusion criteria for the WPW group included being clinically stable, having no history of exercise-induced syncope, no pacemaker, and no recent medication changes that could affect their clinical status. For the control group, age- and sex-matched participants without any chronic illnesses or known risk factors were recruited using a non-probabilistic, convenience sampling approach. Healthy siblings or relatives of patients attending the pediatric cardiology clinic were invited to participate. In addition, participants were recruited through online announcements and parent groups. Some healthy children who presented to the outpatient clinic for routine health certificates required for sports participation or school activities were also invited. All controls were screened to ensure they met the inclusion and exclusion criteria and were not receiving any treatment or follow-up for cardiovascular or chronic conditions.

Exclusion criteria applied to both groups. Participants were excluded if they had neurological or genetic disorders, congenital, structural or ischemic heart disease, or orthopedic or cognitive impairments that could interfere with test performance. Additionally, individuals who had received regular physical activity counseling or participated in a structured exercise training program within the past year were excluded from the study.

### Procedure

**Fig. 1:**
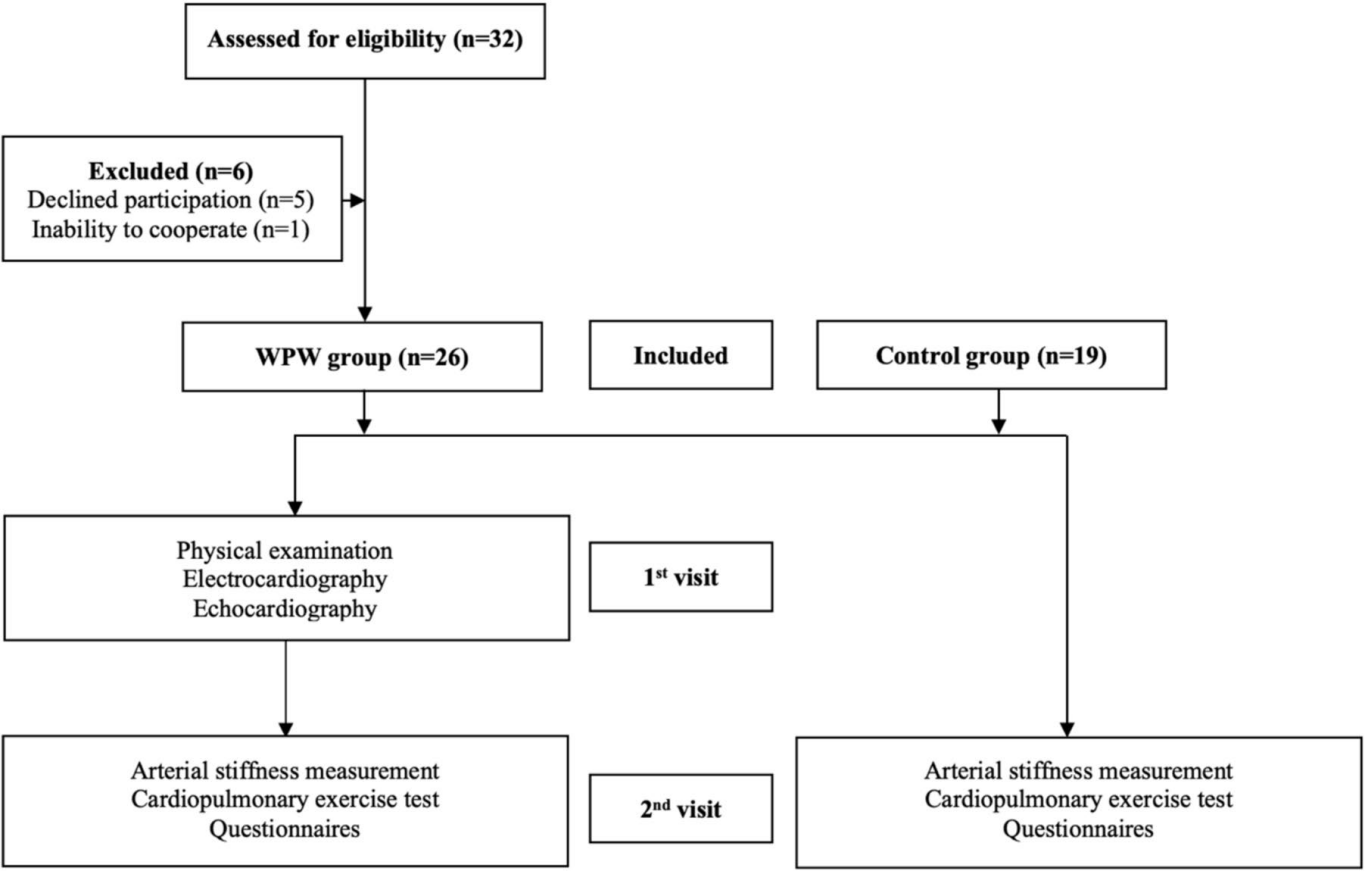
The study flowchart (Abbreviation: WPW, Wolff-Parkinson-White)

Participants underwent a preliminary evaluation by a pediatric cardiologist to determine study eligibility. This evaluation included a standard clinical examination, participant/parent interviews, electrocardiography (ECG), and imaging. Subsequently, arterial stiffness measurements were conducted on a separate morning. A CPET was then performed by a pediatric cardiologist and a cardiorespiratory physiotherapist. Participants also completed questionnaires assessing physical activity levels, expectations, perceived family support, and parental barriers (Fig. 1). All questionnaires had established Turkish validity and reliability, with permissions obtained from the developers.

### Measurements

#### Clinical characteristics

Clinical characteristics, including age, gender, height, and weight, were recorded for all participants. Height and weight were measured using a stadiometer and electronic scale, respectively. For the WPW group, additional data were collected, including symptoms, medications, ablation history, echocardiographic findings, and electrocardiographic findings.

#### Arterial stiffness

Arterial stiffness was measured non-invasively using the Mobil-O-Graph Pulse Wave Analysis Monitor (IEM, Stolberg, Germany), an oscillometric method employing the ARC Solver algorithm (ARC Solver method, Austrian Institute of Technology). Arterial PWV was calculated by a proprietary algorithm of the device and recorded in m/sec [14, 15]. Additionally, pulse pressure, central systolic blood pressure (cSBP) and augmentation index normalized by 75 bpm (AIx@75) were recorded.

The participants were instructed to abstain from stimulants and physical activity for 24 h prior to measurement [16]. After a 15-min rest period in a quiet, temperature-controlled room, three consecutive measurements were taken with the participant seated and the cuff positioned on the left upper arm [16]. To ensure the robustness of the data, three consecutive measurements were taken, and their mean value, when acceptable, was used for analysis [16, 17].

#### Cardiorespiratory fitness

The CPET was performed on a treadmill ergometer (Cosmed Quark CPET, Rome, Italy) using a modified Bruce protocol with breathe-by-breathe method expired gas analysis. Measured variables included peak oxygen uptake (VO₂_peak_), VO₂ at the ventilatory anaerobic threshold (VAT), oxygen uptake efficiency slope (OUES), respiratory exchange ratio (RER), minute ventilation (VE) to carbon dioxide production (VCO₂) slope, peak heart rate (HR_peak_), and oxygen pulse (VO₂/HR) [18]. VAT (VT1) and the respiratory compensation point (VT2) were identified using the V-slope method [18]. The maximal effort was defined as peak HR > 85% of predicted HR_peak_ (220-age) and/or RER > 1.0, with exhaustion due to dyspnea or fatigue [19, 20]. Continuous 12-lead ECG monitoring was conducted during the test. Blood pressure, oxygen saturation (SpO₂), dyspnea, leg fatigue, and overall fatigue (modified Borg scale) were recorded at rest, every 3 min during exercise, and during recovery [18].

#### Physical activity level

Physical activity levels were assessed using the Physical Activity Questionnaire for Older Children (PAQ-C). [21]. The questionnaire consists of ten items, with nine used to calculate an activity score. Item 10 evaluates whether the child can maintain normal activities despite illness or other interventions in the past week, but it is excluded from the activity score calculation. The PAQ-C includes an activity checklist (14 activities, 5-point scale) and questions regarding activity during specific times (5-point scale). The overall PAQ-C score (1–5) was calculated as the average of items 1–9, with higher scores indicating greater activity [21].

#### Physical activity related expectations

Physical activity outcome expectancies were assessed using the Physical Activity Outcome Expectancies Child Survey (17 items, 3-point Likert scale). The survey includes negative (9 items, score 9–27) and positive (8 items, score 8–24) outcome expectancy subdimensions. Lower scores on the negative dimension indicate less perceived impact of negative effects, while higher scores on the positive dimension indicate greater expected positive effects [22]. For example, in cases where physical activity level is high, positive expectations are low, and negative expectations are high, the individual is likely satisfied with the positive outcomes of physical activity. However, they believe that any discomfort experienced during physical activity would not serve as a barrier to engaging in it.

#### Physical activity related family support perceptions

Each child’s physical activity related family support perception was assessed using Physical Activity Home Environment Child Survey. The survey consists of 5 items. Each “Almost never” response is awarded 1 point, each “Sometimes” response is awarded 2 points, and each “Almost always” response is awarded 3 points. The total score for the scale ranges from 5 to 15. A higher score indicates greater support within the family for the child to be physically active [22].

#### Parents related physical activity barriers

Each parent’s physical activity barriers were assessed using the Physical Activity Barriers Parent Survey. This instrument evaluates a range of personal and environmental factors that may hinder physical activity participation. The survey includes 11 items, grouped into two subscales: items 1–6 assess Social/Environmental Barriers, while items 7–11 assess Personal/Individual Barriers. Responses are scored on a scale from 1 to 5, reflecting the perceived severity of each barrier. Specifically, “Not a barrier” is scored as 1, “Somewhat a barrier” as 3, and “Very much a barrier” as 5. If a response falls between two options, intermediate scores are assigned (2 or 4 points). Higher subscale scores indicate greater perceived barriers to physical activity. Separate total scores are calculated for each subscale [22].

### Statistical analysis

All statistical analyses were performed using IBM SPSS Statistics for Windows, Version 26.0 (Armonk, NY: IBM Corp.). Continuous variables were reported as mean (95% confidence interval) or median (min–max), and categorical variables as frequencies and percentages. Data normality was assessed using the Shapiro–Wilk test and graphical methods. Group comparisons were performed using independent *t*-tests or Mann–Whitney *U* tests, and chi-square tests for categorical variables. Correlations were assessed using Pearson or Spearman coefficients. Changes during CPET were analyzed using the Friedman test with post-hoc Wilcoxon signed-rank tests and Bonferroni correction. A significance level of 0.05 was considered for all analyses. Effect sizes for the Mann–Whitney *U* test were calculated with *r* = z/√n, with interpretations of *r* = 0.1 (small effect), *r* = 0.3 (medium effect), and *r* = 0.5 (large effect) [23].

## Results

Age, sex, and body composition parameters were comparable between the groups (*p* > 0.05). Clinical characteristics of the WPW group are presented in Table 1.

**Table 1:** Clinical characteristics of participants

Variables	WPW (*n* = 26)	Control (*n* = 19)	*p*^a,b,^*
Age (years)	11.12 (95% CI: 9.94–12.29)	12.31 (95% CI: 11.05–13.56)	0.130^a^
Gender			
Female	7 (26.90)	6 (31.60)	0.734^b^
Male	19 (73.10)	13 (68.40)	
Height (cm)	150.34 (95% CI: 142.93–158.34)	158.60 (95% CI: 150.81–166.39)	0.124^a^
Weight (kg)	47.54 (95% CI: 41.11–56.09)	51.67 (95% CI: 45.30–58.04)	0.413^a^
BMI (kg/m^2^)	19.80 (95% CI: 19.04–22.28)	20.49 (95% CI: 18.97–22.01)	0.875^a^
Symptoms			
Palpitation without documented SVT	3 (11.53)	N/A	N/A
Chest pain	1 (3.84)	N/A	N/A
Syncope	1 (3.84)	N/A	N/A
Basic ECG findings			
PR interval (ms)	101 (24.50)	120 (40)	0.001*^,b^
QRS duration (ms)	104 (30)	78 (7)	0.003*^,b^
Medications			
Sotalol	1 (3.84)	N/A	N/A
Metaprolol	1 (3.84)	N/A	N/A
Propafenon	2 (7.69)	N/A	N/A
Propranolol	4 (15.39)	N/A	N/A

The data was summarized using mean (95% CI) or *n* (%)

*WPW* Wolff-Parkinson-White, *CI* confidence interval, *BMI* body mass index, *ECG* electrocardiogram, *SVT* supraventricular tachycardia, *IQR* interquartile range, *NA* not applicable

*p < 0.05

^a^Independent samples test

^b^Mann-Whitney test

Relative VO₂_peak_, HR_peak_, and HR reserve were significantly lower in the WPW group compared to the control group (*p* < 0.05, Table 2). No significant differences were observed in other CPET indices, including absolute VO₂_peak_, OUES, RER, VE/VCO₂ slope, or minimum SpO₂ (*p* > 0.05, Table 2).

**Table 2:** Cardiopulmonary exercise test, arterial stiffness, and physical activity outcomes results of participants

Variables	WPW (*n* = 26)	Control (*n* = 19)	p*^,a,b^	Effect size (r/d)
Cardiopulmonary exercise test				
Peak VO_2_ (ml/kg/min)	24.24 (95% CI: 21.81–26.51)	28.37 (95% CI: 25.20–31.55)	0.031*	0.671
RER	1.13 (95% CI: 1.09–1.16)	1.13 (95% CI: 1.09–1.16)	0.793^a^	0.105
OUES/kg (ml/min/l/min)	30.80 (95% CI: 29.03–36.63)	32.87 (95% CI: 24.88–36.73)	0.519^a^	0.188
VE/VCO_2_ slope	28.35 (18.90–48.70)	27.20 (23.80–41.30)	0.945^b^	0.010
Minimum SpO_2_ (%)	95.33 (95% CI: 94.89–95.74)	95.57 (95% CI: 93.87–95.28)	0.721^a^	0.194
Peak HR (bpm)	177.12 (95% CI: 172.03–182.76)	186.94 (95% CI: 183.99–189.90)	0.005*^,a^	0.918
Peak HR (%)	84.33 (95% CI: 81.69–87.10)	89.21 (95% CI: 87.92–90.49)	0.004*^,a^	0.910
HHR (bpm)	36.04 (95% CI: 29.66–41.45)	24.89 (95% CI: 22.18–27.60)	0.004*^,a^	0.976
Peak VO_2_/HR (ml/beat)	5.60 (2.90–13.30)	7.40 (3.80–12.20)	0.485^b^	0.105
Arterial stiffness measurement				
Pulse pressure	49.80 (95% CI: 46.48–53.72)	46.00 (95% CI: 39.49–52.50)	0.239^a^	0.387
cSBP (mmHg)	99.50 (90.00–125.00)	103.50 (80.00–129.00)	0.212^b^	0.188
AIx@75 (%)	32.00 (9.00–55.00)	27.50 (19.00–52.00)	0.307^b^	0.154
PWV (m/s)	4.47 (95% CI: 4.23–4.51)	4.60 (95% CI: 4.33–4.87)	0.329^a^	0.316
PAQ-C score (Child)	2.87 (95% CI: 2.51–3.31)	3.10 (95% CI: 2.56–3.76)	0.097^a^	0.266
Physical activity outcome expectancies (Child)				
Negative	24.00 (20.00–27.00)	24.00 (15.00–27.00)	0.842^b^	0.031
Positive	10.00 (7.00–16.00)	9.00 (8.00–17.00)	0.105^b^	0.253
Physical activity home environment score (Child)	11.22 (95% CI: 10.30–12.22)	11.26 (95% CI: 9.92–11.92)	1.000^a^	0.021
Physical activity barriers score (Parent)				
Environmental	14.27 (95% CI: 12.08–16.64)	13.26 (95% CI: 10.49–15.07)	0.684^a^	0.216
Personal	13.61 (95% CI: 11.19–16.06)	13.89 (95% CI: 11.70–17.01)	0.673^a^	0.057

The data were summarized using mean (95% CI) or median (min–max)

*WPW* Wolff-Parkinson-White, *CI* confidence interval, *VO*_*2*_ oxygen uptake, *METs* metabolic equivalents, *RER* respiratory exchange ratio, *OUES* oxygen uptake efficiency slope, *VE* minute ventilation, *VCO*_*2*_ carbon dioxide production, *SpO*_*2*_ oxygen saturation, *HR* heart rate, *HRR* heart rate reserve, *cSBP* central systolic blood pressure, *AIx@75* augmentation index normalized by 75 bpm, *PWV* pulse wave velocity, *PAQ-C* physical activity questionnaire for older children

*p < 0.05

^a^Independent samples test

^b^Mann-Whitney test

**Fig. 2:**
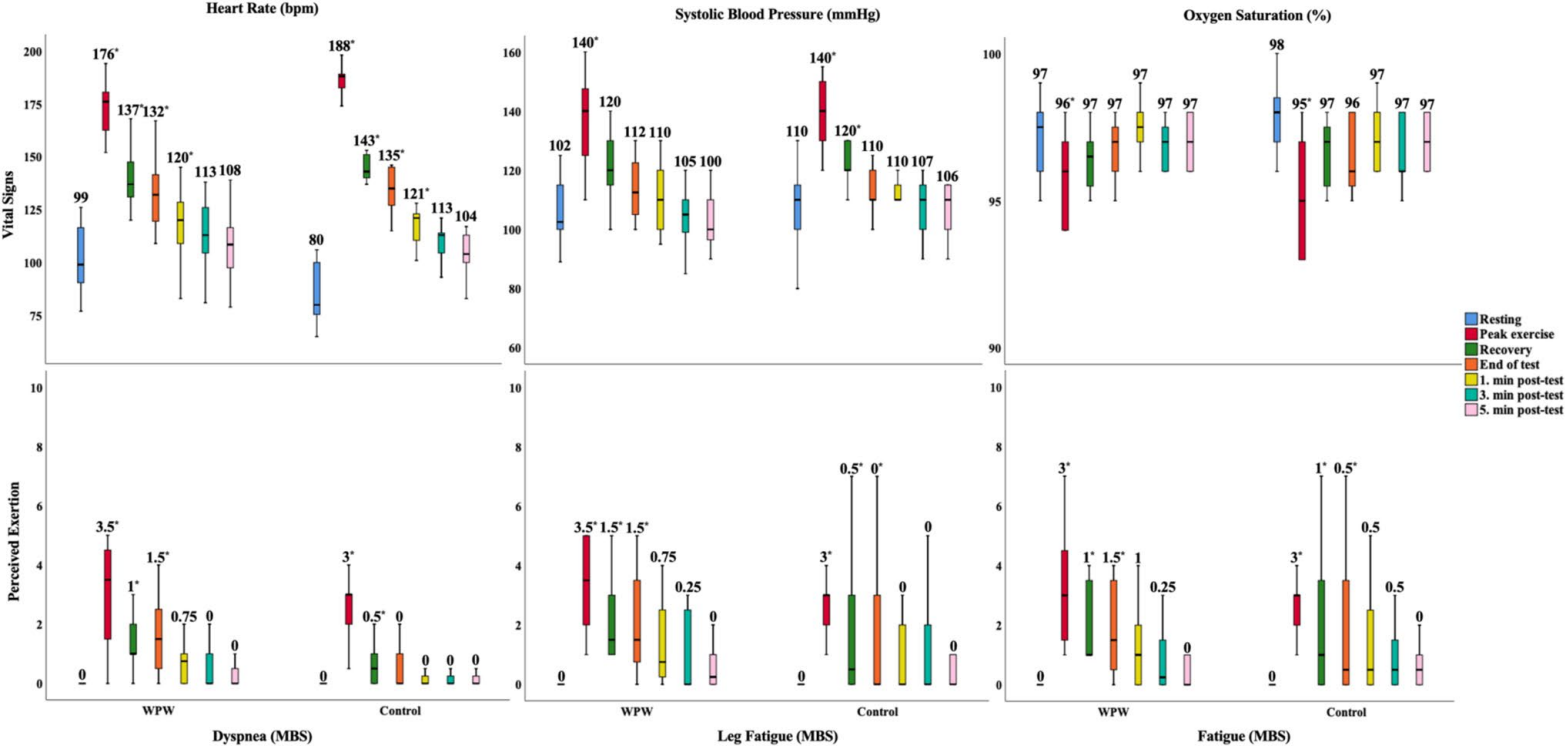
The changes in vital signs and perceived exertion during CPET (Abbreviation: MBS, modified Borg scale. *p < 0.05. A statistically significant difference was observed between the value at resting and at the given stage. The median value for each vital sign was displayed at the top of each bar)

During CPET, resting HR differed significantly from values at peak exercise, recovery, and post-test stages in both groups (*p* < 0.05, Fig. 2). SBP, SpO₂, and perceived exertion returned to baseline levels by the end of recovery, with no group differences (*p* > 0.05), except for baseline and HR_peak_ (*p* < 0.05, Table 1). No adverse events occured during CPET. Only three patients had a prior history of documented tachycardia, all of whom were symptom-free for at least 6 months. Two additional patients reported lifetime symptoms such as palpitations but were asymptomatic at the time of testing. No tachycardia episodes occurred during CPET, and no patient exhibited exertion-induced accessory pathway refractoriness. All patients remained in a pre-excited state throughout the test. None had undergone ablation or were scheduled for imminent procedures at the time of testing.

Arterial stiffness parameters, including pulse pressure, cSBP, AIx@75, and PWV, were comparable between WPW group and control group (*p* > 0.05, Table 2).

Physical activity scores (PAQ-C) trended lower in the WPW group (*p* = 0.097), while outcome expectancies and family support scores showed no significant differences. Parent-reported environmental and personal barriers were also similar across groups (*p* > 0.05, Table 2).

**Fig. 3:**
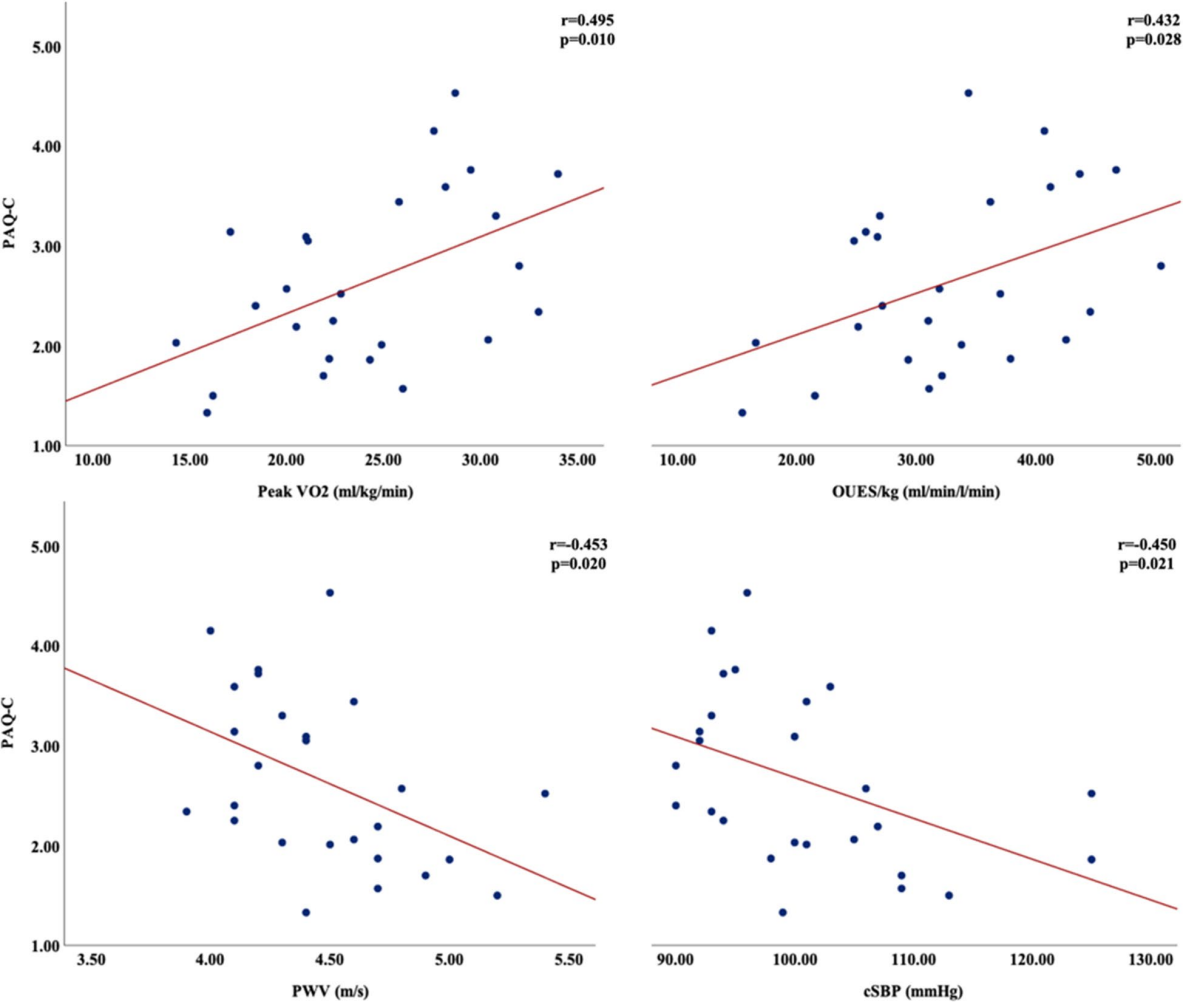
The relationships between physical activity and CPET and arterial stiffness outcomes. (Abbreviation: PAQ-C, physical activity questionnaire for older children; VO_2_, oxygen uptake; OUES, oxygen uptake efficiency slope; PWV, pulse wave velocity; cSBP, central systolic blood pressure; CPET, cardiopulmonary exercise test)

**Fig. 4:**
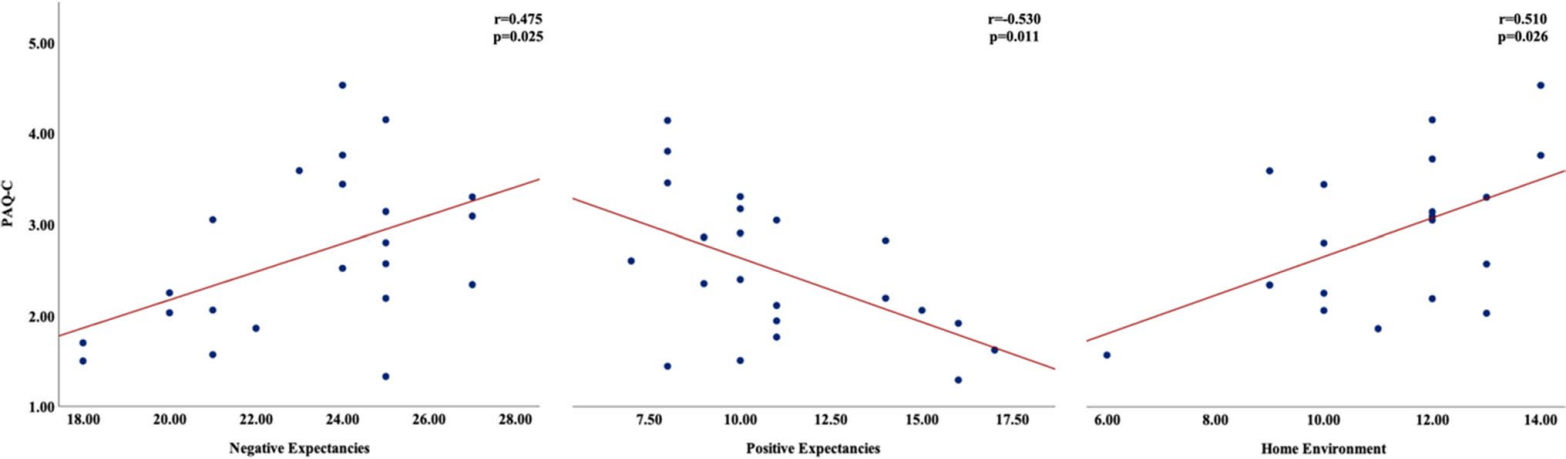
The relationships between physical activity and outcome expectancies and home environment. (Abbreviation: PAQ-C, physical activity questionnaire for older children)

In the WPW group, PAQ-C score correlated moderately with relative VO₂_peak_ (*r* = 0.495, *p* = 0.010), OUES (*r* = 0.432, *p* = 0.028), PWV (*r* =  − 0.453, *p* = 0.020), and cSBP (*r* =  − 0.450, *p* = 0.021) (Fig. 3). Additionally, PAQ-C was positively associated with “Negative Outcome Expectancies” (*r* = 0.475) and “Home Environment” (*r* = 0.510), and negatively with “Positive Outcome Expectancies” (*r* =  − 0.530) (all *p* < 0.05, Fig. 4).

## Discussion

This study is the first to investigate and compare physical activity levels in pediatric WPW patients and their healthy peers. Our findings suggest a trend towards lower physical activity levels in WPW patients, as indicated by PAQ-C scores, potentially linked to impaired exercise capacity or arterial stiffness.

The health benefits of regular physical activity during childhood and adolescence are well-established, including improvements in cardiorespiratory fitness, musculoskeletal strength, bone health, and metabolic function [24, 25]. Children with chronic conditions who meet physical activity recommendations tend to have better fitness, motivation, and confidence [26].

International guidelines advise at least 60 min of moderate-to-vigorous physical activity daily for adolescents [24, 25]; however, many fail to meet these targets, particularly those with chronic illnesses, who face both physiological limitations and psychosocial barriers such as parental overprotection and fear of cardiac events [27–29]. Consequently, lower physical activity levels are observed in children with inherited cardiac diseases compared to healthy peers [30]. A study on pediatric patients with pacemakers found that they had decreased physical activity levels and lower physical fitness [4], while those with long QT syndrome may have comparable activity levels to controls [31]. In our study, although WPW patients showed a trend toward lower physical activity scores, the difference was not statistically significant. This suggests that disease burden and clinical complexity may influence physical activity engagement in pediatric cardiac populations. Nonetheless, encouraging safe physical activity remains essential. Current recommendations emphasize individualized assessment, including ECG, cardiac imaging, and, if indicated, electrophysiological testing or ablation [32]. Yet, there is a lack of targeted strategies for promoting physical activity in children with WPW. Future studies should focus on identifying appropriate exercise types and intensities for this group.

Our study found lower VO₂_peak_ values in WPW patients compared to controls, consistent with previous reports in children with pre-excitation [33]. This reduced cardiorespiratory fitness may be linked to exercise-related conduction behavior. Patients with persistent pre-excitation during exercise tended to show more impaired CPET results than those in whom pre-excitation diminished [33], suggesting that sustained pre-excitation may interfere with chronotropic or hemodynamic responses. Accessory pathway location may also play a role, as it can affect conduction properties and sympathetic response [34]. Disappearance of pre-excitation during exercise has been associated with longer refractory periods and lower arrhythmic risk, while persistent pre-excitation may reflect less adaptive conduction [34]. Delta wave behavior depends on the balance between sympathetic drive, pathway refractoriness, and AV nodal conduction. At higher heart rates, pre-excitation may disappear in left-sided pathways but persist in septal ones, potentially affecting ventricular efficiency [34]. These patterns stem from the fundamental disruption of cardiac conduction caused by the accessory pathway, leading to dyssynchronous myocardial activation and reduced stroke volume and oxygen delivery [35].

Additionally, lower HRpeak and HRR observed in our cohort may reflect chronotropic insufficiency, possibly influenced by beta-blocker or antiarrhythmic use in over 30% of patients. These findings further support the notion that while physical activity levels may not always differ significantly, the physiological limitations imposed by the underlying cardiac condition can still impact overall fitness and exercise capacity. Future research should explore myocardial and conduction factors contributing to reduced fitness in WPW, including analyses by pathway location and delta wave response.

Arterial stiffness is a clinically significant marker of vascular damage and a strong predictor of cardiovascular disease (CVD) [36]. Although arterial stiffness is strongly associated with ageing and is clinically evident in older individuals, it can also occur in early childhood, in what is known as the preclinical phase [37]. Arterial stiffness, assessed through parameters like PWV, cSBP, and AIx@75, is an important indicator of cardiovascular health, especially in populations with specific cardiac conditions such as WPW syndrome [38]. In our study, arterial stiffness outcomes in WPW patients were comparable to those of healthy controls, which may be partly attributed to the use of beta-blockers or antiarrhythmic medications—agents known to affect vascular tone and reduce arterial stiffness in hypertensive patients [39, 40]. However, data on their specific effects in WPW are limited and warrant further research. Notably, we observed moderate negative correlations between physical activity levels and arterial stiffness parameters in WPW patients. This suggests that higher physical activity may enhance arterial compliance in this population. These findings are consistent with broader evidence linking physical activity to improved vascular health through mechanisms such as reduced inflammation, enhanced endothelial function, and vascular remodeling [41, 42].

Although most adolescents recognize the importance of regular physical activity, factors such as low motivation, time constraints, safety concerns, cost, self-confidence, and personal habits often limit their participation [43]. These barriers may be intensified in youth with cardiovascular conditions due to additional physiological and psychosocial challenges. In WPW patients, further obstacles—such as limited access to facilities, parental overprotection, and fear of exertion—may further reduce activity levels. In this study, the physical activity outcome expectancies and family support levels of patients with WPW, as well as their parents’ perceived barriers to physical activity, were found to be similar to those of their healthy peers. Interestingly, the relationship between PAQ-C scores and physical activity outcome expectancies suggests a nuanced dynamic: pediatric WPW patients who perceive the negative consequences of physical activity as less significant tend to be more active. Conversely, those with higher expectations for positive outcomes from physical activity report lower activity levels. This pattern implies that children who are already physically active may find the perceived benefits of exercise sufficient, while requiring stronger negative effects for these to act as a deterrent. These findings highlight the complex interplay between physiological limitations and psychosocial factors in shaping physical activity behaviors in pediatric WPW patients, emphasizing the need for targeted interventions to enhance engagement in physical activity.

### Limitations and implications

This study had limitations, including a relatively small sample size, which limited ability to perform subgroup analyses. Future studies with larger cohorts should aim to examine the influence of key factors such as gender, accessory pathway location, symptomatic versus asymptomatic presentation, treatment status (receiving vs. not receiving antiarrhythmic therapy), and age group differences. Analyzing these variables in more homogeneous subgroups may help to better understand the specific physiological and psychosocial profiles of pediatric WPW patients. Additionally, using objective methods for measuring physical activity and employing qualitative mixed-methods approaches could provide more comprehensive data.

Despite these limitations, this study provides valuable insights into physical activity and related factors in pediatric WPW patients. This study provides preliminary insights that may contribute to the development of targeted guidelines and highlights the need for structured physical activity counseling and intervention strategies in this specific population.

## Conclusion

Although physical activity levels, expectations, family support, and parental barriers were similar between WPW patients and controls, lower cardiorespiratory fitness in WPW patients may increase the risk of reduced physical activity. Our findings also suggest a link between physical activity, exercise capacity, and vascular health, emphasizing the need to consider both physiological and psychosocial factors in managing physical activity in pediatric WPW patients.

## Data Availability

No datasets were generated or analysed during the current study.

## Abbreviations

AIx@75: Augmentation index normalized by 75 bpm
CPET: Cardiopulmonary exercise testing
cSBP: Central systolic blood pressure
ECG: Electrocardiogram
HR: Heart rate
OUES: Oxygen uptake efficiency slope
PAQ-C: Physical Activity Questionnaire for Older Children
PWV: Pulse wave velocity
RER: Respiratory exchange ratio
SpO₂: Oxygen saturation
VAT: Ventilatory anaerobic threshold
VCO₂: Carbon dioxide production slope
VE: Minute ventilation
VO₂: Oxygen uptake
WPW: Wolff-Parkinson-White
